# Non-invasive Multimodality Imaging of Coronary Vulnerable Patient

**DOI:** 10.3389/fcvm.2022.836473

**Published:** 2022-02-24

**Authors:** Marjorie Canu, Alexis Broisat, Laurent Riou, Gerald Vanzetto, Daniel Fagret, Catherine Ghezzi, Loic Djaileb, Gilles Barone-Rochette

**Affiliations:** ^1^Department of Cardiology, University Hospital, Grenoble Alpes, Grenoble, France; ^2^Univ. Grenoble Alpes, INSERM, CHU Grenoble Alpes, LRB, Grenoble, France; ^3^French Alliance Clinical Trial, French Clinical Research Infrastructure Network, Paris, France; ^4^Department of Nuclear Medicine, University Hospital, Grenoble Alpes, Grenoble, France

**Keywords:** vulnerable plaque, vulnerable patient, coronary artery disease, multimodal imaging, chronic coronary syndrome, risk stratification

## Abstract

Atherosclerotic plaque rupture or erosion remain the primary mechanism responsible for myocardial infarction and the major challenge of cardiovascular researchers is to develop non-invasive methods of accurate risk prediction to identify vulnerable plaques before the event occurs. Multimodal imaging, by CT-TEP or CT-SPECT, provides both morphological and activity information about the plaque and cumulates the advantages of anatomic and molecular imaging to identify vulnerability features among coronary plaques. However, the rate of acute coronary syndromes remains low and the mechanisms leading to adverse events are clearly more complex than initially assumed. Indeed, recent studies suggest that the detection of a state of vulnerability in a patient is more important than the detection of individual sites of vulnerability as a target of focal treatment. Despite this evolution of concepts, multimodal imaging offers a strong potential to assess patient's vulnerability. Here we review the current state of multimodal imaging to identify vulnerable patients, and then focus on emerging imaging techniques and precision medicine.

## Introduction

Coronary artery diseases (CAD) remain the largest single cause of death in the World. Traditionally, atherosclerosis management consists in detecting obstructive CAD and ischemia. However, this paradigm is being challenged as revascularization of obstructive CAD failed to reduce acute coronary events in recent studies (1, 2) and most of these events occur on non-obstructive plaques (3). Novel imaging techniques have emerged in this setting, targeting vulnerable coronary plaques that are more likely to lead to a plaque thrombosis and an acute coronary syndrome (ACS). However, the prospective follow-up of vulnerable plaques is deceiving in predicting future coronary events (4) and the mechanism of plaque thrombosis seems to be more complex, where not only plaque progression, but also systemic parameters such as inflammation, thrombogenic and dynamic change processes are intricated, so that the concept of vulnerable patients was introduced. Moreover, while rupture of thin-cap fibro atheroma (TCFA) remains the main cause of acute coronary events (55–65%), plaque erosion (30–35%) an, to a lower extend microcalcifications (2–7%), are also known to be responsible for such events through distinct pathobiological mechanisms (5). Importantly, most plaque with thrombosis are clinically silent and lead to plaque progression and luminal stenosis (6). Vulnerable patients, in whom the thrombosis of a vulnerable plaque is likely to result in a clinical event in the future, are not only characterized by vulnerable plaques, but also vulnerable blood and vulnerable myocardium (7). We review here current and in-development non-invasive techniques, based on multimodal imaging on this field.

## Anatomic Features

Identifying high-risk plaques before ACS occurs has been a major research goal. Retrospective studies analyzed progression of CAD among patients presenting with ACS, by comparing plaque features on previous coronary angiography exams. Most coronary acute events occurred on unobstructed lesion at baseline (8). The histological study of culprit plaques, responsible for ACS, helped identifying common underlying features in high-risk plaques. Some of these features can be identified with invasive imaging techniques (9) but cannot be translated to routine clinical practice because of costs, and due to the fact that invasive techniques such as optical coherence tomography (OCT) or intravascular ultrasound (IVUS) cannot be employed in large populations and are restricted to patient previously identified at high risk. Improvement of multimodal imaging techniques of the plaque allow non-invasive visualization of such features.

### Vulnerable Coronary Plaque

Computed tomography coronary angiography (CTCA) that permits precise visualization of the plaque became a first-line diagnostic test in the assessment of suspected CAD. At the simplest level, the segment involvement score (SIS) sums the number of diseased coronary segments, whilst the stenosis severity score (SSS) also incorporates a weighting factor for stenotic severity (10). CTCA, with high spatial resolution scanners, can provide precise structural information of the coronary artery wall and can assess for the presence and constituents of atherosclerotic plaque even in the absence of flow limiting disease. Based on histological analysis, TCFA are mainly characterized by a large necrotic core, thin fibrous cap (<65 mm), inflammation (predominantly in the form of macrophage infiltration), angiogenesis, plaque hemorrhage, positive remodeling and microcalcification (11). Not all of these features can be evaluated using non-invasive imaging. However, a number of morphologic criteria that can be assessed using CTCA have been employed to identify such lesions. In a SCOT-Heart *post-hoc* analysis, the presence of vulnerable plaque features such as positive remodeling, low attenuation plaque, spotty calcification, and the “napkin ring” sign were validated against intravascular invasive imaging (12). The results of PROMISE (13) and SCOTHEART studies (12) confirmed the association between adverse plaque characteristics and outcomes. Obstructive coronary disease is also a major risk predictor, and the combination of adverse plaque features with obstructive disease appears to confer the greatest risk (13). Moreover, CT- Leaman score, which combines stenotic severity, myocardium at risk, and high-risk plaque features, allows an improved risk stratification of the plaque (14). Currently, while they appear to be less competitive than CTCA for the identification a vulnerable plaque, a number of other high-resolution imaging systems can also be employed. Table 1 describes the imaging modalities, their strengths and limitations and a comparison between the modalities in assessing the different aspects that characterize a vulnerable patient. Cardiac magnetic resonance imaging (CMR) holds the great advantage to be not only a non-invasive, but also a non-ionizing imaging technique. Black blood sequences confer a fairly good spatial resolution of the coronary wall (15) allowing detection of adverse plaque features, such as positive remodeling, plaque hemorrhage and subclinical thrombus (16). However, such approaches have largely been limited to the visualization of the main proximal vessels, because of the reduced spatial resolution, as compared to CTCA. Furthermore, CMR is a time-costly and less available imaging technique. Trans-thoracic echocardiography does not allow the precise visualization and analysis of coronary arteries. However, ultrasound enables carotid plaques characterization, such as differentiation between artery occlusions and high-grade stenosis, plaque morphology (plaque surface, flow data) and plaque neovascularization, thereby enabling to estimate its vulnerability (17).

**Table 1:** Non-invasive multimodal imaging assessment of vulnerable plaques and patients in chronic coronary syndrome.

**Imaging modalities**	**Strenghts**	**Limitations**
**Morphological imaging techniques**		
CTCA	High spatial resolution. Fast and good availability. Plaques: high risk features Measure of coronary artery disease burden on the whole coronary tree.	Limited by calcifications, stents. Radiation, contrast.Limited temporal resolution
CACS	Fast and good availability, low cost. No contrast, low radiation. Coronary atherosclerotic burden	Limited spatial resolution. No detection of non-calcified plaques.
CMR	Radiation free. Not limited by calcifications?	Poor spatial resolution. Costly, less available, Duration. Contraindications: Claustrophobia, metallic devices.
TTE	No radiation, fast, low cost, availability	No precise visualization of CA
**Molecular imaging techniques**		
PET	Molecular imaging of Inflammation, microcalcification activity, Thrombogenicity by Targeted radionucleotides.	Poor temporal and spatial resolution, radiation costly, duration, limited availability, FDG myocardial uptake.
SPECT	SPECT tracers are relatively inexpensive in comparison of PET agents. More available than PET.	Poor spatial and temporal resolution Radiation, costly, duration Less tracer available than PET in the field of CA.
CMR	Nanoparticles: Gd-DTPA, USPIO	Clinical translation to aortic and carotid atherosclerosis. Moderate diagnostic accuracy in coronary arteries
CTCA	FAI disponible by all 64 slice CTCA	indirect inflammation assessment Cost
TTE	CEUS: targeted microbubbles in preclinical studies	Technical challenges for clinical translation
**Local hemodynamic forces**		
CTCA	Wall shear stress: CT CFD	In development
**Myocardial function and tissue characterization**		
CMR	Reference for cardiac function assessment. Tissue characterization: fibrosis	Cost, availability ECG gating necessary
TTE	Cardiac systolic and diastolic function. Fast, low cost, availability.	No tissue characterization
CT	Cardiac volumes and function Tissue characterization: fibrosis	Retrospective acquisition: radiation Contrast injection ECG gating necessary Performance for tissue characterization is still average
PET/SPECT	Left ventricular systolic function	Poor temporal and spatial resolution ECG gating necessary

*CTCA, Computed tomography coronary angiography; FFR, fractional flow reserve; CFD, computational flow dynamics; CACS, Coronary artery calcium score; CMR, Cardiac magnetic resonance imaging; CA, coronary arteries; PET, positron emission tomography, FDG, fluorodeoxyglucose; SPECT, single photon emission computed tomography; Gd-DTPA, gadolinium-diethylenetriaminepentaacetic acid; USPIO, ultrasmall superparamagnetic iron oxide; FAI, fat attenuation index; CEUS, Contrast enhanced ultrasound*.

If CTCA remains the best non-invasive imaging technique to detect coronary plaques and assess their vulnerability, the prospective follow-up of these vulnerable plaques is deceiving in predicting future coronary events (4) which remain low in this population. Indeed, while being of high negative predicting value, the positive predictive value of identifying a high-risk plaque in large cohort studies such as SCOTHEART or PROMISE was found to be low, with only ~5% of events at 5 years. There are several explanations for this low positive prognostic value. The first being that the presence of at least one lesion with vulnerable plaque characteristics is probably not as rare as might have been assumed. In addition, the occurrence of an acute event does not only require the presence of a vulnerable plaque but also that of other parameters such as prothrombotic factors. Therefore, a plaque can rupture without being symptomatic. Moreover, atherosclerotic lesions are characterized by dynamic evolution, and it is not excluded that vulnerable plaques pacifies over time (18).

### Coronary Atherosclerosis Disease Burden

Imaging techniques measuring coronary atherosclerosis disease burden, or call also “the adverse plaque burden”, therefore confer a better risk stratification for future cardiovascular events at the patient level. While it has been shown that the more vulnerable coronary plaques a patient has, the greater the likelihood of major adverse cardiovascular events (MACE), it is rarely the plaques identified as vulnerable that will be responsible for acute arterial thrombosis. This highlights the fact to switch from a focus on individual lesions to atherosclerotic disease burden for coronary artery disease risk assessment (19). Coronary artery calcium (CAC) is a non-invasive, rapid computed-tomography (CT) technique that quantifies atherosclerotic calcifications, a well-described process occurring as a healing response to pathological inflammation within the plaque. CAC scoring is a direct marker of CADB for each patient and is effective in predicting the risk of future atherosclerotic cardiovascular events in asymptomatic patients (20). A large observational study involving 25,253 patients in the United States with a mean follow-up of 6.8 years showed that survival varied significantly according to the extent of CAC. Indeed, survival rates varied from 99.4 to 87.8%, respectively, for CAC scores of 0 and >1,000 (*p* < 0.0001) (21). However, while CAC enables estimating plaque burden, macrocalcification are not restricted to vulnerable lesions but also occurs in more stable lesions, so that more specific parameters are needed. The quantification of CADB by measure of the number of vulnerable plaques on the entire coronary tree has great potential. However, quantify CADB across the coronary vasculature is challenging. This is now possible in a rapid and robust fashion with semiautomatic software by certain vulnerable plaque features. Recently, low attenuation plaque burden appears as a strong predictor of fatal or non-fatal myocardial infarction irrespective coronary artery calcium score (22). The development of these software improving the reproducibility also allow to observe the evolution of coronary atherosclerosis disease burden under treatment (23). CTCA is a key tool for identifying high risk patients, by anatomic features. However, coupling anatomic data with molecular imaging may improve risk stratification for patient's vulnerability assessment.

## Factor of Dynamic Plaque Change

Coronary atherosclerosis presents a dynamic nature and plaques with at least one vulnerable feature are in fact relatively common and appear dynamic process of formation and healing. Identifying the factors associated with an adverse dynamic plaque change is therefore a major priority. Molecular imaging has the enormous advantage of allowing the visualization, characterization and quantification of biological processes. Even though the molecular imaging potential of MRI and ultrasound is being investigated (24–26), nuclear imaging represents the most mature modality in this perspective. Several traceable physio pathological processes associated to adverse dynamic plaque change toward vulnerable patient could be use.

### Inflammation

Atherosclerosis is an immuno-inflammatory illness powered by lipids. The major role of inflammation in the development of coronary artery plaques and in the pathophysiology of plaque rupture was comforted by the results of emerging studies in which colchicine, an anti-inflammatory treatment, was associated with a reduction in ischemic events after a MI (27) and in patients with chronic coronary disease (28). Nuclear molecular imaging, by tracking inflammation with specific molecular targets, allows the direct visualization of inflammation within the plaque. Imaging modalities include CT- positron emission tomography (PET), CMR-PET and CT-single photon emission computed tomography (SPECT). Known tracers include ^18^F-fluorodeoxyglucose (^18^F-FDG), ^68^Gallium(^68^Ga-DOTATATE), and ^68^Ga-Pentixafor. In CAD, ^18^F-FDG reflects plaque inflammation by detecting glucose uptake in regions of high metabolic activity (29). Hybrid ^18^F-FDG PET- CT allow precise anatomic identification of coronary plaques coupled with molecular inflammatory inflammation. This hybrid imaging technique showed increased inflammatory activity of perivascular adipose tissue adjacent to coronary arteries segments with plaques (30) and correlation between ^18^F-FDG PET imaging and histological macrophage uptake of carotid plaques after carotid endarterectomy (31). In ACS patients, metabolic activity detected by this radiotracer is identified not only in the culprit lesion, but also in other atherosclerotic site, such as ascending aorta or left main coronary artery, showing atherosclerotic vulnerability at the patient level (32). However, coronary ^18^F-FDG lacks cell specificity and signal can be obscured by background myocardial uptake. In atherosclerotic plaque tissue, CXCR4 expression might be used as a surrogate marker for inflammatory atherosclerosis. *In vivo* use of ^68^Ga-Pentixafor appear feasible to evaluation of CXCR4 expression in human carotid atherosclerotic lesions (33). ^68^Ga-DOTATATE binds to the somatostatin receptor subtype-2 (SST2) found on the surface of pro-inflammatory M1 macrophages and targets inflammation. It was validated using PET-CT imaging in patients with carotid plaques, in carotid plaques showing high-risk CT features, and in culprit coronary plaques in the setting of ACS with superior coronary imaging and excellent macrophage specificity (34).

**Figure 1:**
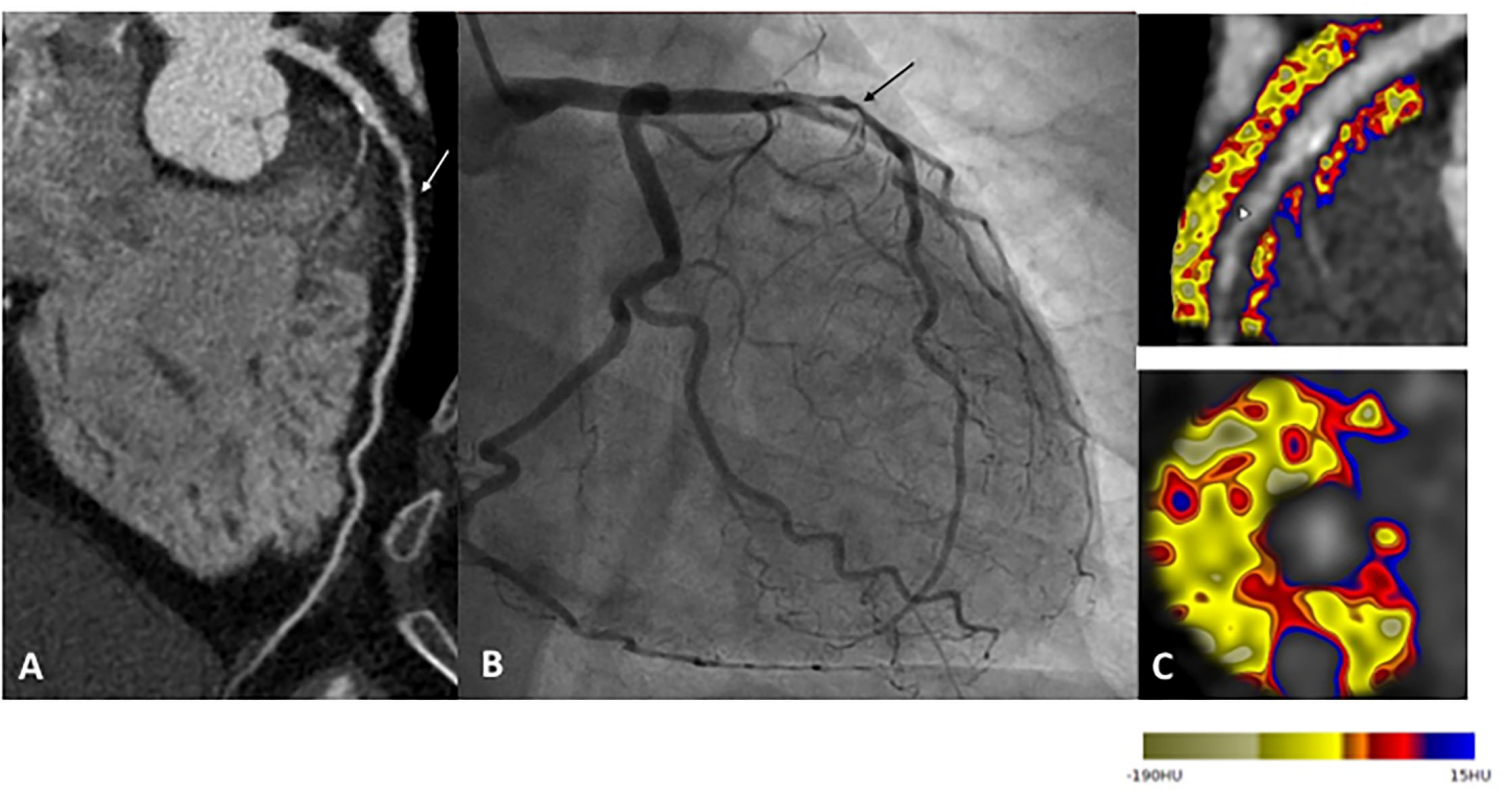
Example of a patient reporting exertional dyspnea. In **(A)**, CTCA showed significant CAD on left anterior descending (LAD) artery (white arrow), classified CAD-RADS 4A. Coronary angiogram confirm severe stenosis of proximal LAD (black arrow), angioplasty followed by stenting was performed to relieve symptoms **(B)**. Finally, CTCA post-treatment based on the FAI-Score values **(C)** on three arteries, the coronary atherosclerotic plaque burden and the clinical risk factors showed low CaRi-Heart Risk, thereby predicting low risk of future acute coronary events and permitted treatment goals and follow-up strategies personalization.

Off line post processing of CTA datasets proved to be very useful for the analysis of the complex interactions between coronary arteries and perivascular adipose tissue are complex. Adverse perivascular adipocyte profile, associated with some metabolic conditions, is known to trigger pro-inflammatory changes within coronary arteries (35). Recent fundamental studies suggest a bidirectional relationship between perivascular fat and coronary arteries. Coronary inflammation inhibits lipid accumulation in adjacent adipocytes, resulting in a gradient in the lipid content of perivascular fat. The CTCA analysis of epicardial and pericoronary fat provides information to improve plaque and patient ischemic risk stratification, with CTCA-measured epicardial fat volume being associated with CAD and cardiovascular events (36). However, the prognostic implications of epicardial fat attenuation remain controversial (37, 38). These discordant results could reflect the heterogeneity of epicardial fat composition and support the hypothesis that inflammatory changes in perivascular fat might be a local process limited to the adjacent regions of vulnerable plaques. CTCA-derived fat attenuation index (FAI), using the Cari-Heart algorithm, indirectly quantifies arteries inflammation burden, by analyzing the signal from perivascular fat (Figure 1). Higher FAI values correspond to adipose tissue morphologic changes associated with coronary inflammation (39). Recent *post-hoc* analyses of prospective CTCA and outcome data showed the incremental prognostic value of FAI to detect high-risk plaques (HRP), beyond traditional risk factors (40) and beyond HRP plaque features (41).

### Microcalcification Activity

Preclinical and clinical evidence show that calcification is one of the body's primary responses to injury. ^18^F-NaF is a marker of microcalcification activity, which binds with high affinity to the exposed surface of hydroxyapatite, a key mineral component of vascular calcification and detects plaque microcalcification, another feature of vulnerable plaques. Increased ^18^F-NaF uptake is observed in coronary plaques that show multiple adverse features on CT, on virtual histology (VH)- IVUS, and on OCT (42) and could improve the risk stratification of patients with CAD.

### Thrombogenicity

Several studies used radionuclide imaging approaches to analyze several thrombosis-related molecular markers (43). Factor XIIIa radiotracer and (44) ^18^F-GP1 are safe and promising novels PET tracer for imaging acute arterial thrombosis with a favorable biodistribution and pharmacokinetic profile (45). However, none is yet available in clinical practice.

### Local Hemodynamic Forces

Although the anatomic and chemical features of potentially vulnerable plaques play a significant role, additional information regarding dynamic plaque change may provide significant information. Among hemodynamic-associated biomechanical forces that increase plaque vulnerability, special attention has been paid to wall shear stress (WSS) (46). WSS may be assessed using CTCA through sophisticated post processing based on computational fluid dynamics and shows that high wall shear stress had an incremental value over luminal narrowing in discriminating high-risk plaques (47).

## Future directions

Identification of the vulnerable patient remains a challenge for cardiology today which partly depends upon progresses performed by imaging modalities. High temporal and spatial resolution of anatomical modalities is a prerequisite considering the small size and motion of coronary arteries. Cardiac hybrid imaging allows to obtain complementary morphological and molecular features information in a single setting. CT-SPECT and CT-PET are widely used, and CMR-PET may represent an alternative. However, CT-SPECT or CT-PET imaging are also controversial because of radiation dose issues. Due to technological progress, the most recent high-pitch scanning protocols using dual-source CT scanners have lowered doses into the sub-milli-Sievert range. Safety and dosimetry now represent important elements to be taken into account in the development of any radionuclide. This notion of low irradiation is essential for the repetition of the examinations during the follow-up. With regards to technological progress, SPECT detector with cadmium-zinc-telluride (CZT) improve count sensitivity, system resolution, and energy resolution, enabling significant reductions in administered activities or acquisition time, as well as facilitating dynamic SPECT. A multitude of single-photon emitters is available with half-lives longer than those of commonly used PET radionuclides, facilitating their distribution to more remote centers. In addition, SPECT tracers are relatively inexpensive in comparison of PET agents. However, the constant evolution of PET-CT and SPECT-CT technology makes it challenging to use equipment combining the latest technological developments in SPECT, PET (48) or CT. For example, there is no hybrid machine with the latest evolution of spectral photon-counting CT (49). In this setting, the development of image fusion software may represent an alternative by obviating the need for a hybrid machine combining the latest innovations (50). This process will be supported by a shift from specializing in a particular technique that is applied to multiple organ imaging, to a cardiovascular-based approach in which the diagnostic expert is more concerned with the integration of results into clinical decision-making, and the impact of diagnostic imaging on clinical outcomes.

Images often contain more information than what is comprehensible by visual inspection. The current development of radiomics, whereby voxel-level information is extracted from digital images and used to derive multiple numerical quantifiers of shape and tissue character, may address this potential. For example, coronary CTA radiomics may provide a more accurate tool to identify vulnerable plaques compared with conventional methods (51). It is important that one keeps in mind that modalities scans are more than plain images; they are data. The analysis of such data using artificial intelligence is currently revolutionizing medical imaging (52). Big data include enormous numbers of predictors and outcomes with complex non-linear links, and conventional statistics usually fail to analysis them. Accordingly, machine-learning algorithms frequently use recently developed statistical program. Machine learning combining clinical and CCTA data was found to predict 5-year all cause of mortality significantly better than existing clinical or CCTA metrics alone (53). Machine learning can combine a large amount of data from imaging, but also from biomarkers, genomics and proteomics to derive the most accurate risk stratification models (54). The real revolution for imaging is deep learning. Deep-learning use multiple layers of convolutional neural networks (CNNs) which learn how to extract the most relevant data of the image and how best to combine them to acute event. Unlike radiomic, CNN can automatically discover such relationships at the pixel level without being defined before-hand based on human knowledge (55). Deep-learning is a promising method to develop future software incorporating further automation techniques of CADB, would therefore help facilitate more wide spread clinical adoption.

**Figure 2:**
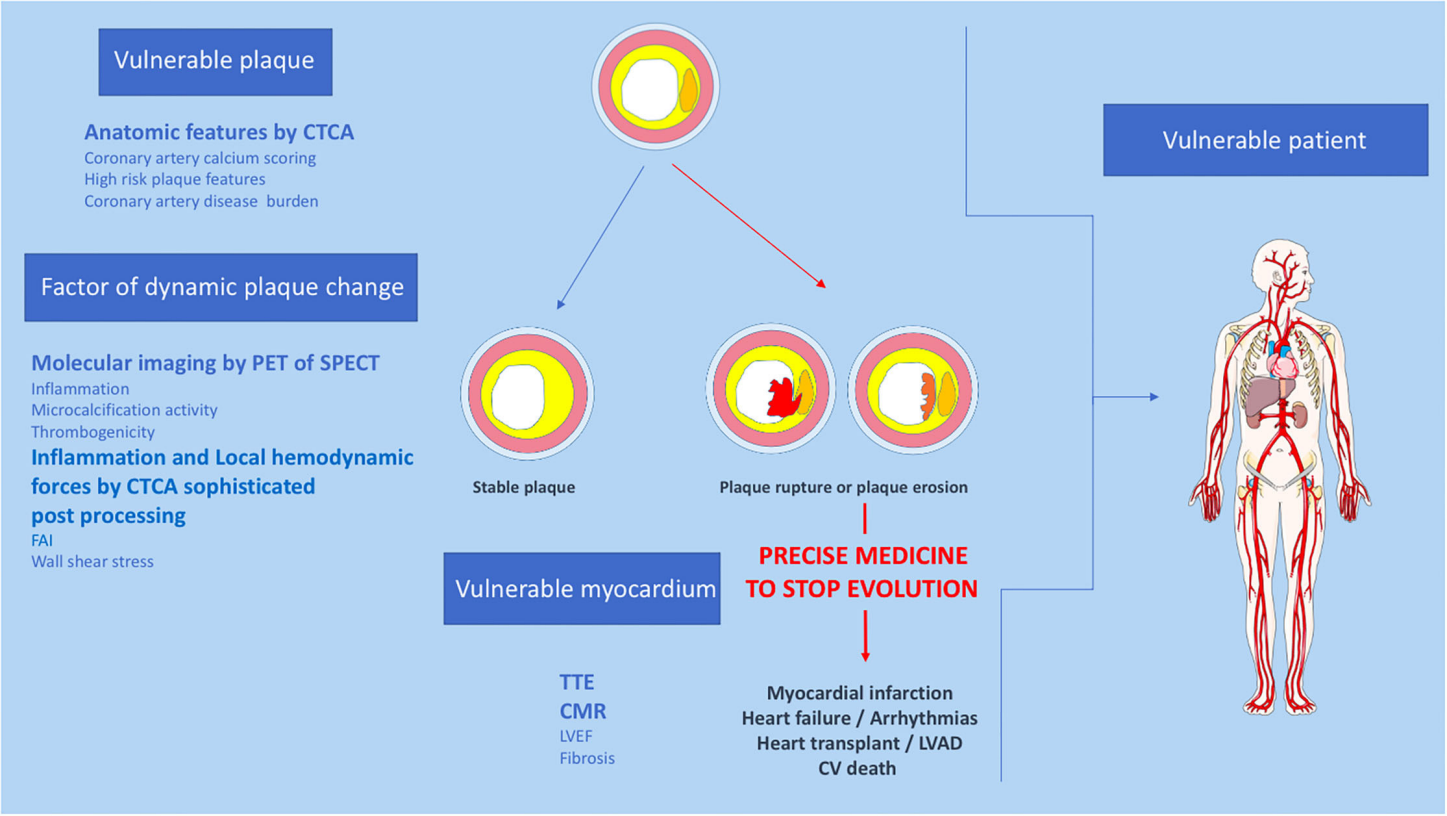
Proposition of non-invasive multimodality imaging strategy to detect and treat coronary vulnerable patient. CTCA, Computed tomography coronary angiography; CV, cardiovascular; CMR, cardiac magnetic resonance imaging, FAI, fat attenuation index; LVEF, left ventricular ejection fraction; PET, positron emission tomography; SPECT, single photon emission computed tomography; TTE, trans thoracic echocardiography.

Above all, the identification of vulnerable patients should lead to precision medicine (Figure 2). One should not try to predict MACE but rather to develop strategies that identify patients at risk of developing MACE who require individualized drug management. Non-invasive imaging modalities aim to address this need, but such methods need to be widely available, safe, accurate, and ultimately cost-effective in order to ensure a meaningful impact on healthcare and patient outcomes. Despite its attractivity for the identification of vulnerable patients, multimodal imaging has a cost. A screening strategy must therefore be developed in parallel with imaging. It might be based upon the careful examination of clinical characteristics such as traditional risk factors and cholesterol levels and then use diagnostic test simple and available (i.e., CAC). Patients at high risk could be referred for screening by multimodality imaging techniques.

## Conclusion

Imaging of vulnerable coronary plaque features has advanced greatly over the past decade and has improved our understanding of the highly complex and dynamic nature of coronary atherosclerosis. Despite the many advances in cardiovascular imaging, the prediction of atherosclerotic plaque rupture responsible for myocardial infarction remains difficult and is not applicable in clinical practice. However, multimodal imaging, in particular CT and nuclear molecular imaging, allow the identification of major characteristics of the vulnerable patient. Finally, randomized studies using these technological innovations will allow us to move toward precision medicine.

## Author Contributions

MC, LD, and GB-R contributed to conception and design of the mini review. MC wrote the first draft of the manuscript. AB, LR, and GB-R wrote sections of the manuscript. All authors contributed to manuscript revision, read, and approved the submitted version.

## Conflict of Interest

The authors declare that the research was conducted in the absence of any commercial or financial relationships that could be construed as a potential conflict of interest.

## Publisher's Note

All claims expressed in this article are solely those of the authors and do not necessarily represent those of their affiliated organizations, or those of the publisher, the editors and the reviewers. Any product that may be evaluated in this article, or claim that may be made by its manufacturer, is not guaranteed or endorsed by the publisher.
